# Reproductive stage affects the daily behavioral patterns of parthenogenetic marbled crayfish

**DOI:** 10.1093/cz/zoaf062

**Published:** 2025-09-17

**Authors:** Koushik Das, Paride Balzani, Davinder Kaur, Jan Kubec, Miloš Buřič, Antonín Kouba, Marek Let

**Affiliations:** Faculty of Fisheries and Protection of Waters, South Bohemian Research Centre for Aquaculture and Biodiversity of Hydrocenoses, University of South Bohemia in České Budějovice, Zátiší 728/II, Vodňany 389 25, Czech Republic; Faculty of Fisheries and Protection of Waters, South Bohemian Research Centre for Aquaculture and Biodiversity of Hydrocenoses, University of South Bohemia in České Budějovice, Zátiší 728/II, Vodňany 389 25, Czech Republic; Faculty of Fisheries and Protection of Waters, South Bohemian Research Centre for Aquaculture and Biodiversity of Hydrocenoses, University of South Bohemia in České Budějovice, Zátiší 728/II, Vodňany 389 25, Czech Republic; Faculty of Fisheries and Protection of Waters, South Bohemian Research Centre for Aquaculture and Biodiversity of Hydrocenoses, University of South Bohemia in České Budějovice, Zátiší 728/II, Vodňany 389 25, Czech Republic; Faculty of Fisheries and Protection of Waters, South Bohemian Research Centre for Aquaculture and Biodiversity of Hydrocenoses, University of South Bohemia in České Budějovice, Zátiší 728/II, Vodňany 389 25, Czech Republic; Faculty of Fisheries and Protection of Waters, South Bohemian Research Centre for Aquaculture and Biodiversity of Hydrocenoses, University of South Bohemia in České Budějovice, Zátiší 728/II, Vodňany 389 25, Czech Republic; Faculty of Fisheries and Protection of Waters, South Bohemian Research Centre for Aquaculture and Biodiversity of Hydrocenoses, University of South Bohemia in České Budějovice, Zátiší 728/II, Vodňany 389 25, Czech Republic

**Keywords:** feeding behavior, freshwater crayfish, laboratory model, locomotion, reproduction, shelter

## Abstract

Behavioral patterns are complex physiological and ethological phenomena usually synchronized with the day–night cycle. Reproduction is an important modulator of these activities in crayfish females with differences between non-reproductive and egg-carrying individuals. However, the behavioral pattern of maturing females with forming glair glands has never been assessed. Hence, we compared the behavioral patterns of non-reproductive, egg-carrying, and glair glands females of marbled crayfish *Procambarus virginalis*, observing their behavior in an empty arena and in the presence of critical resources—shelter and food. Crayfish were video-recorded for 24 h and their activity, distance moved, and velocity analyzed. The results indicated distinct behavioral patterns linked to reproductive stages and resource availability. Specifically, non-reproductive females displayed higher activity levels than egg-carrying and glair glands females, which spent most of their time in the shelter. On the other hand, the presence of food increased the overall activity of glair glands compared with egg-carrying females and reduced shelter usage, highlighting a trade-off between foraging and safety. These findings demonstrated an interaction between the reproductive stage and resource acquisition behavior, providing insights into crayfish adaptive strategies and, in the context of invasive crayfish, potential impacts on ecosystem dynamics. This study highlights the importance of accounting for the reproductive stage and timing in experiments involving marbled crayfish or other crayfish species.

## Graphical Abstract

**Graphical Abstract:**
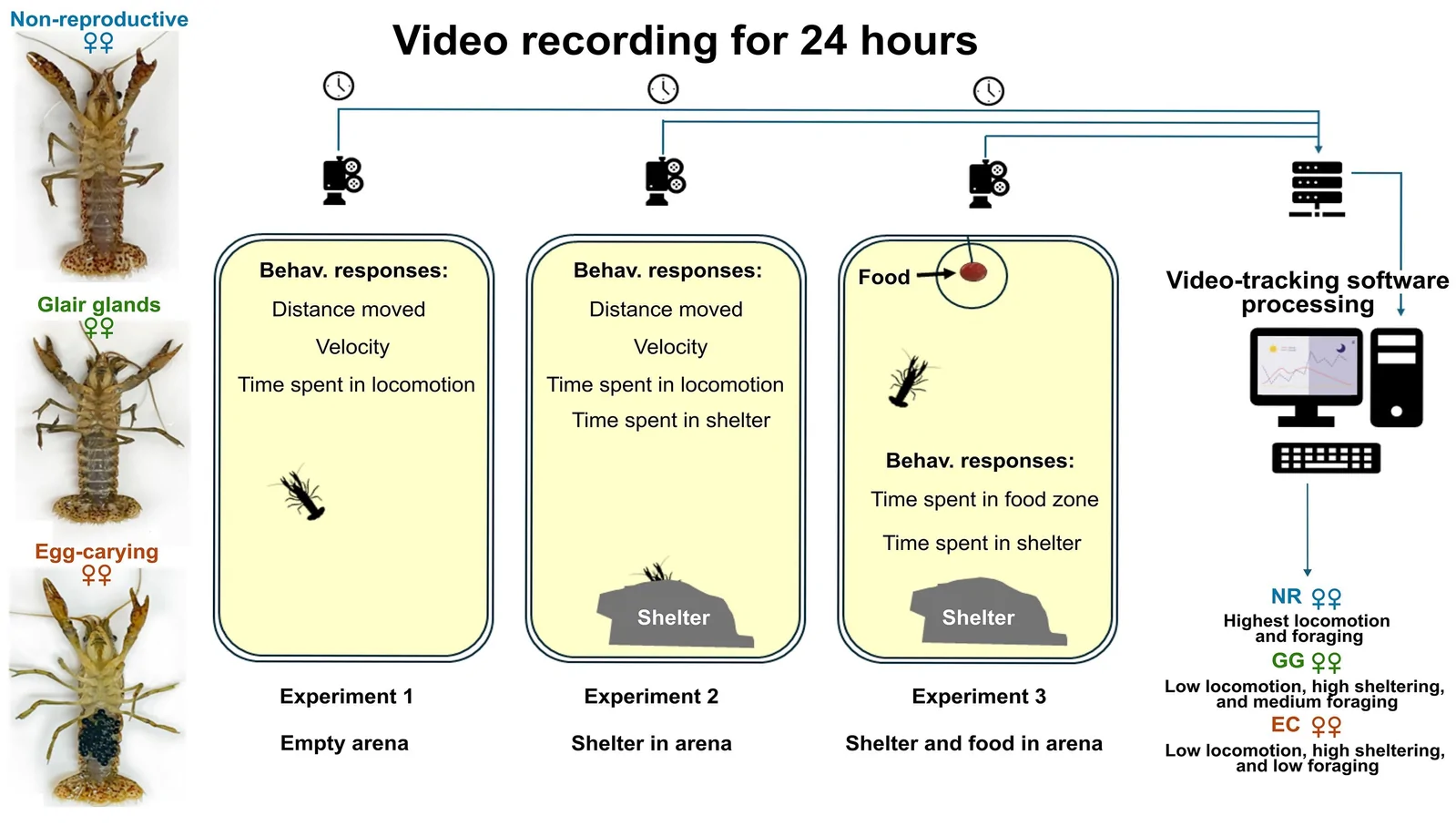
fx1

Animal daily behavioral patterns are complex physiological and behavioral phenomena usually synchronized with the day–night cycle (Aschoff 1965; Vitaterna et al. 2001; Hut et al. 2012). Indeed, animals can be identified as primarily nocturnal (predominantly active at night) or diurnal, active during daytime (Fanjul-Moles et al. 1996; Fanjul-Moles and Prieto-Sagredo 2003; Farca Luna et al. 2010). Plasticity in their daily behavioral patterns (Smarr et al. 2013) is associated with the energy budget (Helm et al. 2017), but also with various behavioral traits, such as aggressiveness, sociability, or a solitary lifestyle, and influences survival in the environment (Strauss and Dircksen 2010). Thus, by allocating time to various behaviors in response to intrinsic and extrinsic factors, behavioral patterns enable animals to adopt behavioral strategies that ultimately optimize fitness by minimizing risks and maximizing the foraging and reproductive success (Kronfeld-Schor and Dayan 2003; Kronfeld-Schor et al. 2013; Helm et al. 2017).

Crayfish are known to adopt flexible behavioral patterns (Westin and Gydemo 1988; Hazlett 1994; Gherardi et al. 2000, 2002; Fero and Moore 2008). They are recognized as ecologically important organisms in diverse habitats, including lotic and lentic freshwater ecosystems (Usio and Townsend 2002; Reynolds et al. 2013) and as such they have also been used as suitable model animals in various disciplines, including ecology, neurophysiology, and ethology (Vogt 2008; Thomas et al. 2016; Hossain et al. 2018; Toutain et al. 2025). In terms of behavioral studies, several activity assays have been established using crayfish to study, for instance, burrowing (Guo et al. 2020), sheltering (Hossain et al. 2021), feeding activities (Reisinger et al. 2021; Soto et al. 2023), and exploration (de Abreu et al. 2020). Crayfish exhibit a broad spectrum of behavioral patterns, with most species showing nocturnal activities (Aquiloni et al. 2005; Musil et al. 2010; Daněk et al. 2019), although some species display diurnal activity (Bubb et al. 2002; Barbaresi et al. 2004). Furthermore, it has been documented that crayfish can alter their behavioral patterns in different contexts. For example, the red swamp crayfish *Procambarus clarkii* (Girard, 1852) is largely nocturnal, but in the laboratory its activity mostly occurred during daytime (Gherardi et al. 2000). Seasonal variations have also been reported for juveniles of the noble crayfish *Astacus astacus* (Linnaeus, 1758) under natural light and with shelter, with a nocturnal activity in summer and autumn and a diurnal activity in winter (Westin and Gydemo 1988).

The marbled crayfish *Procambarus virginalis* Lyko, 2017 is a recently evolved parthenogenetic freshwater crayfish species, first detected in the aquarium pet trade ∼25 years ago (Vogt 2008). It is a triploid and obligate parthenogenetic species derived from the sexually reproducing diploid slough crayfish *Procambarus fallax* (Hagen, 1870) (Gutekunst et al. 2021; Maiakovska et al. 2021). This species is a generalist omnivore (Lipták et al. 2019; Veselý et al. 2021; Balzani et al. 2025) and was identified as an invasive species of EU concern (EU Regulation No. 1143/2014 and Commission Implementing Regulation No. 2016/1141), threatening native crayfish populations as well as freshwater ecosystems via its prolific, all-female monoclonal population-producing capability and wide tolerance to environmental conditions (Veselý et al. 2015, 2017; Kouba et al. 2021). The marbled crayfish can live up to ∼3 years and has also been shown to successfully co-exist with other invasive species (Vogt 2011; Weiperth et al. 2020). Considering its prolific life history traits (e.g., rapid growth and maturation, frequent reproduction, high fecundity) and reproduction mode resulting in clonality, the marbled crayfish has been proposed as a laboratory model species for multiple purposes (Martin et al. 2007; Vogt 2011; Hossain et al. 2018).

In crayfish species, studies have shown that egg-carrying females, compared with non-reproductive ones, spend less time feeding and moving, and more time sheltering, thereby protecting their developing eggs and early juveniles (Vogt 2013). Therefore, reproductive females are usually excluded from experiments, especially in behavioral studies (Findlay et al. 2015; Linzmaier and Jeschke 2020). Prior spawning, crayfish females develop prominent whitish glair glands on the ventral side of the pleon producing mucus in which the eggs are fertilized and subsequently attached to the pleopods (Reynolds 2002; Vogt 2015, 2018). In particular, in late winter/spring and late summer/autumn, marbled crayfish individuals with glair glands are frequently observed in the laboratory stocks (Vogt 2015) as well as the wild (Dobrović et al. 2021). Also, given the rapid reproduction of the species, females in different stages of reproduction (from glair gland development to spawning and then egg-carrying) commonly emerge in the test groups (e.g., during exposure tests in ecotoxicology), thus disbalancing experimental setups. To what degree these reproductive stages (i.e., non-reproductive, with developed glair glands, and carrying eggs) influence the behavior of marbled crayfish females and crayfish females in general, is somewhat unknown. In this study, we analyzed the behavioral patterns of marbled crayfish across three different stages (non-reproductive—NR, glair glands-having—GG, and egg-carrying females—EC) under three different experimental conditions most commonly used in laboratory experiments involving crayfish: absence of critical resources (activity in an empty arena), presence of a shelter, and presence of both shelter and food. We hypothesized that the reproductive stage influences daily behavioral patterns in marbled crayfish, particularly in terms of locomotion, shelter use, and feeding behavior. Based on this, we expected NR females to show the highest levels of locomotion and foraging, with minimal use of shelters. In contrast, we expected EC females to be less active in terms of locomotion and spend more time in the shelter. Finally, we expected GG females to show an intermediate behavior between the two other groups, with moderate locomotor activity and shelter use.

## Materials and methods

### Experimental animals

Marbled crayfish were cultured in an indoor laboratory and maintained as a communal stock in a closed recirculating aquaculture system under artificial light with a 14L:10D light:dark regime. Water temperature was maintained at 21 ± 1 °C, dissolved oxygen always stayed above 7.5 mg l^−1^, and pH ranged from 7.3 to 7.8. Crayfish were fed *ad libitum* daily with defrosted chironomid larvae, freshly grated carrots, and a commercial fish diet (Sera Granugreen, Sera, Heinsberg, Germany).

Similar-sized intermolt individuals of marbled crayfish [cephalothorax length (CL) 16–21 mm, Supplementary Table S1] belonging to three groups (NR females, GG females, and EC females with attached eggs) were randomly selected from the source stock and used for the experiments. Individuals with missing or regenerating claws were not chosen for the experiment, as their feeding abilities may be compromised (Soto et al. 2023; Toutain et al. 2025). For each animal used in the experiments, CL was measured using Vernier calipers (accuracy 0.1 mm), and weight (W) using an electronic pocket balance (Kern & Sohn GmbH, Belingen, Germany; accuracy 0.1 g).

### Experimental design

**Figure 1:**
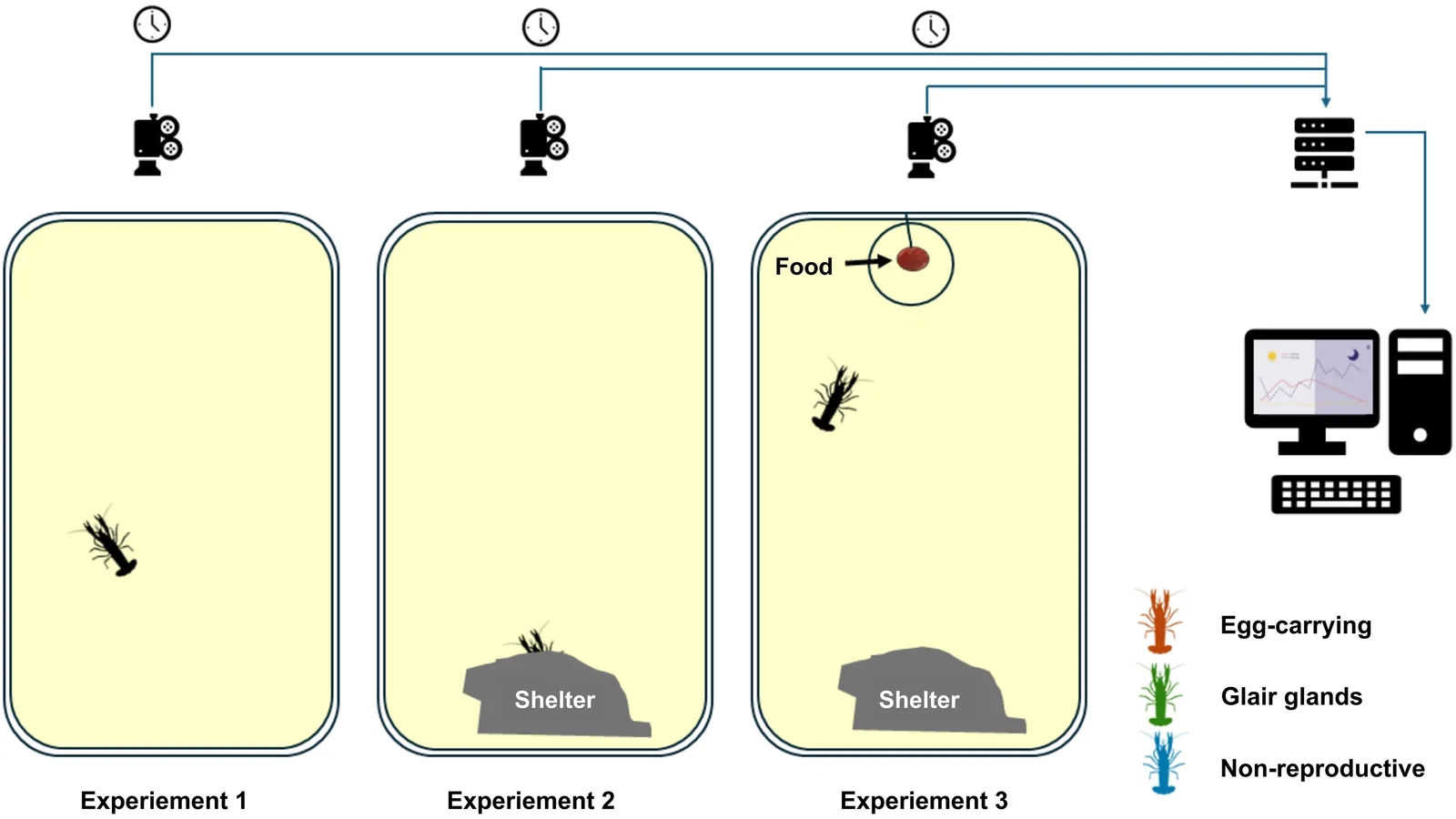
Schematic diagram of the experimental setup for monitoring marbled crayfish (*Procambarus virginalis*) behaviors. The figure illustrates the setup used to monitor and record the behavior of crayfish under three different conditions: (1) an empty arena; (2) an arena with shelter; and (3) an arena with shelter and food. Each condition was recorded using overhead cameras connected to a data acquisition system. The data collected from the cameras was transferred to a computer for analysis and visualization

We conducted three independent laboratory experiments providing distinct conditions to understand how reproductive stages and resource availability interactively determine the daily behavioral patterns of marbled crayfish NR, GG, and EC females (Fig. 1). Experiment 1 aimed at investigating the marbled crayfish daily behavioral patterns in an empty arena. The other two experiments focused on the behavioral patterns of these groups in the presence of critical resources: shelter (Experiment 2) and both shelter and food (Experiment 3).

Before the start of all experiments, crayfish were individually introduced in a rectangular (400 × 285 × 75 mm) plastic arena with 4 L of dechlorinated tap water and a layer of ∼1 cm fine sand (∼1 mm particle size) at the bottom and acclimated for 48 h to minimize the handling effect on the behavioral observations. The position of arenas was randomized across the three groups inside a closed room. During the acclimation period, no water changes were performed to avoid additional disturbance, and a small amount of food was provided (except in Experiment 3) to prevent nutritional stress while maintaining naturalistic conditions. Following acclimation, animals were video-recorded for 24 h (from morning 8 AM to the next morning 8 AM) using a digital video camera (Sony HDR-CX240, Sony, Japan) attached above arenas. Fluorescent tubes provided illumination during the day (daylight, 2,310 lm), and red monochromatic light-emitting diodes were provided (Seed 60, Grow Led, Czech Republic, spectrum 660 nm) to allow recording in the darkness. All the experiments were conducted in complete silence and with minimal disturbance inside the room. After each trial, the animals were removed from the arena, and the water was completely drained, sand rinsed, replaced with clean sand to eliminate any residual chemical cues from previous individuals, and prepared for the subsequent trial.

For Experiment 2 and Experiment 3, a shelter, and a shelter and food, respectively, were provided to the animals in the arenas described above, all the rest being equal to Experiment 1. The shelter used in these experiments was an aquarium opaque cave (10 cm length and 6 cm height) placed in the middle of the short side of the arena, with its opening oriented toward the center. In Experiment 3, food (∼1 g of fresh carp meat-dorsal fillet white muscle) was tied up by a transparent fishing line and fastened to the opposite side of the shelter within the arena. Using a standardized portion of the fish meat helped ensure consistent olfactory cues across trials and minimized variability in crayfish behavioral responses. Although crayfish in the communal stock were maintained on a mixed diet (carrot, chironomid larvae, and fish pellets), fresh fish meat was used during the experiment to ensure it remained anchored within the food zone (Fig. 1), prevent dispersal across the arena, and provide a strong, consistent foraging stimulus standardized across individual observations. All individuals received the same standardized food source as the objective was to test behavioral responses to the presence of a resource, not dietary preference. During the acclimation and all experiments, the water temperature (21 ± 1 °C), and light:dark regime (14L:10D) were maintained.

### Behavior observation and data acquisition

Video footages were continuously recorded for 24 h and analyzed for each hour separately using the EthoVision® XT 15.0 software (Noldus Information Technology, Wageningen, Netherlands) using a multiple-arena module. The following variables were measured: (i) total distance moved (in cm; defined as the cumulative distance traveled by the crayfish per hour based on changes in the center of mass), (ii) mean velocity (in cm s^−1^; calculated as the average speed during periods of detected movement), and (iii) crayfish activity (portion of the time in which crayfish locomotion was detected per each hour; locomotion was considered as any movement exceeding 0.5 cm s^−1^, based on the velocity output from EthoVision XT, which allowed us to differentiate between stationary postures and active displacement). Furthermore, in Experiments 2 and 3, in which the shelter was present (Fig. 1), we also recorded the time spent inside the shelter (per each hour). In Experiment 3, (i) the time spent in the shelter and (ii) the time spent in the food zone (defined as a 2-cm radius circle centered directly on the food—fresh carp meat; Fig. 1) were quantified. The total number of tested animals in each group (NR, GG, and EC) was always balanced at the beginning of the experiments—22 replications in Experiment 1, and 16 replications in Experiments 2 and 3. No significant differences were observed among groups in animals' CL or W across all three experiments (Supplementary Table S1). Experiment 1 was conducted between March and April 2021 during seven recording days, Experiment 2 was conducted in May 2021 during four recording days, and Experiment 3 was also conducted in May 2021 during five recording days. Up to 10 crayfish individuals were recorded during the same day. After each recording, used individuals were released into aquaria housing the recorded crayfish. Individuals were not reused in any experiment. The observations that failed due to technical or biological issues were excluded from the dataset (see Table 1 in *Results* for the final number of crayfish included into the analyses).

**Table 1:** Behavioral types of marbled crayfish (*Procambarus virginalis*) detected by the most parsimonious latent class linear mixed models for each response variable from individual experiments: Exp 1—empty arenas, Exp 2—presence of shelter in arenas, and Exp 3—presence of shelter and food in arenas.

Response variable “*Behavioral pattern characteristics*”	Behavioral pattern	Frequency of occurrence across all groups (%)	Number of individuals/total number of individuals		
			EC	GG	NR
**Exp 1** **empty arena**					
**Distance moved**					
*Day-time locomotion*	1	6.35	1/20	2/21	1/22
*24-h locomotion*	2	93.65	19/20	19/21	21/22
**Velocity**					
*Peak velocity*	1	12.70	2/20	1/21	5/22
*Constant velocity*	2	87.30	18/20	20/21	17/22
**Activity**					
*High activity in the morning*	1	7.94	2/20	2/21	1/22
*Increased activity in the morning and night*	2	82.54	17/20	16/21	19/22
*High activity at night*	3	9.52	1/20	3/21	2/22
**Exp 2** **presence of shelter in arena**					
**Distance moved**					
*Night-time locomotion*	1	80.56	13/14	8/11	8/11
*Day-time locomotion*	2	19.44	1/14	3/11	3/11
**Velocity**					
*Slow and constant velocity*	1	55.56	7/14	3/11	10/11
*Morning and evening peak velocity*	2	16.67	2/14	3/11	1/11
*Velocity reduced day-time*	3	27.77	5/14	5/11	0/11
**Activity**					
*Low activity*	1	72.22	14/14	11/11	1/11
*Activity reduced day-time*	2	27.78	0/14	0/11	10/11
**Time spent in shelter**					
*Sheltering*	1	72.22	14/14	11/11	1/11
*Reduced sheltering*	2	27.78	0/14	0/11	10/11
**Exp 3** **presence of shelter and food in arena**					
**Time spent in shelter**					
*Prominent day-time sheltering*	1	30.95	2/13	4/14	7/15
*Bimodal sheltering*	2	28.57	3/13	5/14	4/15
*Night and morning sheltering*	3	9.52	1/13	1/14	2/15
*Less prominent day-time sheltering*	4	30.95	7/13	4/14	2/15
**Time spent in food zone**					
*Night-time foraging*	1	35.71	1/13	6/14	8/15
*Bimodal day-time foraging activity*	2	19.05	2/13	2/14	4/15
*Consistently low foraging activity*	3	45.24	10/13	6/14	3/15

Abbreviations: EC, egg-carrying females; GG, glair gland females; NR, non-reproductive females.

### Statistical analysis

All statistical analyses were conducted using the R software (ver. 4.1.1; R Core Team 2021). The response variables included: (i) log + 1-transformed distance moved (cm), hereafter “distance moved”; (ii) log + 1-transformed velocity (cm s⁻¹), hereafter “velocity”; (iii) log + 1-transformed time spent in locomotion (s)/3,600, hereafter “activity’—used in Experiments 1 and 2; (iv) log + 1-transformed time spent in shelter (s)/3,600, hereafter “time spent in shelter”—used in Experiments 2 and 3; and (v) log + 1-transformed time spent in the food zone (s)/3,600, hereafter “time spent in food zone” used in Experiment 3. After applying the transformation to reduce heteroscedasticity and improve normality of residuals in the models described below, model assumptions were considered sufficiently met based on visual inspection of regression diagnostics plots (square roots of model residuals plotted against fitted values). Given the relatively large dataset (1,512; 864; and 3,014 records for Experiments 1, 2, and 3, respectively), formal tests of residual normality or homoscedasticity were not applied.

For each experiment and individual marbled crayfish, 24-h time series of the above response variables were first visualized using locally estimated scatterplot smoothing (LOESS) using the *ggplot2* R package (Wickham 2016). To identify dominant behavioral patterns, latent class linear mixed models (LCLMMs) were applied across the 24-h period using the *lcmm* R package (Proust-Lima et al. 2017). LCLMMs including one to five latent classes were compared, and those with an optimal number of latent classes, hereafter referred to as “behavioral patterns', were selected using the Bayesian Information Criterion (BIC) and log-likelihood (LogLik) values. Identified behavioral patterns were assigned to individuals and subsequently used as a covariate in further modeling. In addition, each behavioral pattern was empirically interpreted and named based on its temporal characteristics.

Generalized additive (mixed-effect) models [GA(M)Ms] were then performed using the *mgcv* R package (Wood 2017) to assess temporal dynamics in each response variable. Residual variance was modeled using a normal distribution, and cyclic cubic splines were used to ensure a cyclic daily pattern. The modeling framework tested: (i) whether including individual identity as a random intercept improved GAMs fit; (ii) whether temporal dynamics of the individual response variables differed across reproductive groups (NR, GG, and EC)—after the accounting for the variability explained by behavioral patterns—; and (iii) in the absence of significant interactions, whether groups differed in mean values of response variables or exhibited any overall 24-hour trends. GA(M)Ms were selected based on Akaike Information Criterion (AIC), and comparisons were further supported by likelihood ratio tests (LRTs). Fixed effects and their interactions were also evaluated using LRTs.

## Results

### Experiment 1

**Figure 2:**
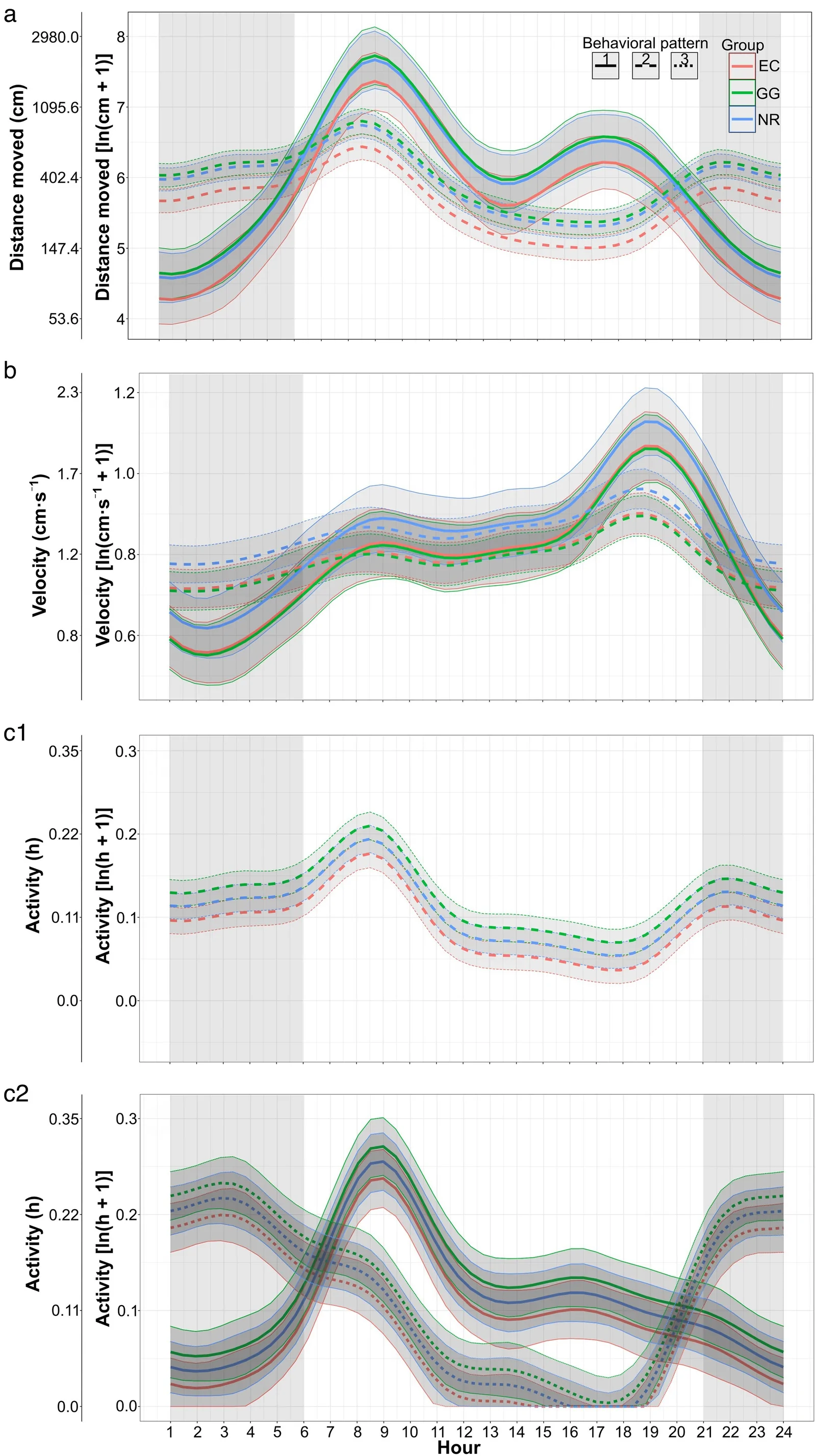
Experiment 1—empty arenas: fixed effects of the most parsimonious generalized additive mixed models (GAMMs) of marbled crayfish (*Procambarus virginalis*) behavioral responses. Distance moved, velocity, and activity were used as response variables in the GAMMs, diplayed in panels a, b, and c, respectively. Panel C is divided into two subplots (C1 and C2) to provide better visualization. Gray ribbons represent 95% confidence regions. Shaded areas represent dark period, normal area light period. The *y*-axes are shown as both raw values and log + 1-transformed. Abbreviations: EC, egg-carrying females; GG, glair gland females; and NR, non-reproductive females. For further details about behavioral patterns see Table 1.

In empty arenas, two distinct behavioral patterns were identified by the most parsimonious LCLMMs for distance moved (Supplementary Table S2 and Figure S1A): (i) “day-time locomotion” and (ii) “24-h locomotion” (Figure 2A). Both behavioral patterns were detected without prevalence in any group (Table 1 and Supplementary Figure S2A). The main effect of group was significant (*χ*^2^_2_ = 13.7, *P* = 0.001) but all groups had the same temporal dynamic (*χ*^2^_3_ = 6.4, *P* = 0.1). EC showed the lowest values of distance moved but GG and NR did not differ substantially (Figure 2A). As for velocity, two behavioral patterns were found (Supplementary Table S2 and Figure S1B): (1) “peak velocity” and (2) “constant velocity”. Peak velocity was attributed to 23% of crayfish individuals in the NR group, while only 5% and 10% were modeled in GG and EC, respectively (Table 1 and Supplementary Figure S2B). The main effect of group was significant (*χ*^2^_2_ = 6.7, *P* = 0.04), but all groups showed the same temporal dynamics (*χ*^2^_3_ = 1.53, *P* = 0.67). NR showed the highest velocity compared to EC with GG (Figure 2B). Using activity as the response, three behavioral patterns were detected: (1) “high activity in the morning”, (2) “increased activity in the morning and night”, and (3) “high activity at night”. All these behavioral patterns were detected without prevalence in any group (Table 1 and Supplementary Figure S2C). Similarly, as in the previous response variables, only the main effect of group was significant (*χ*^2^_2_ = 10.8, *P* = 0.005). The activity of GG and NR was higher compared with EC, especially in the crayfish exhibiting the second most frequent behavioral pattern (high activity at night) (Table 1, Figure 2C1,2).

### Experiment 2

**Figure 3:**
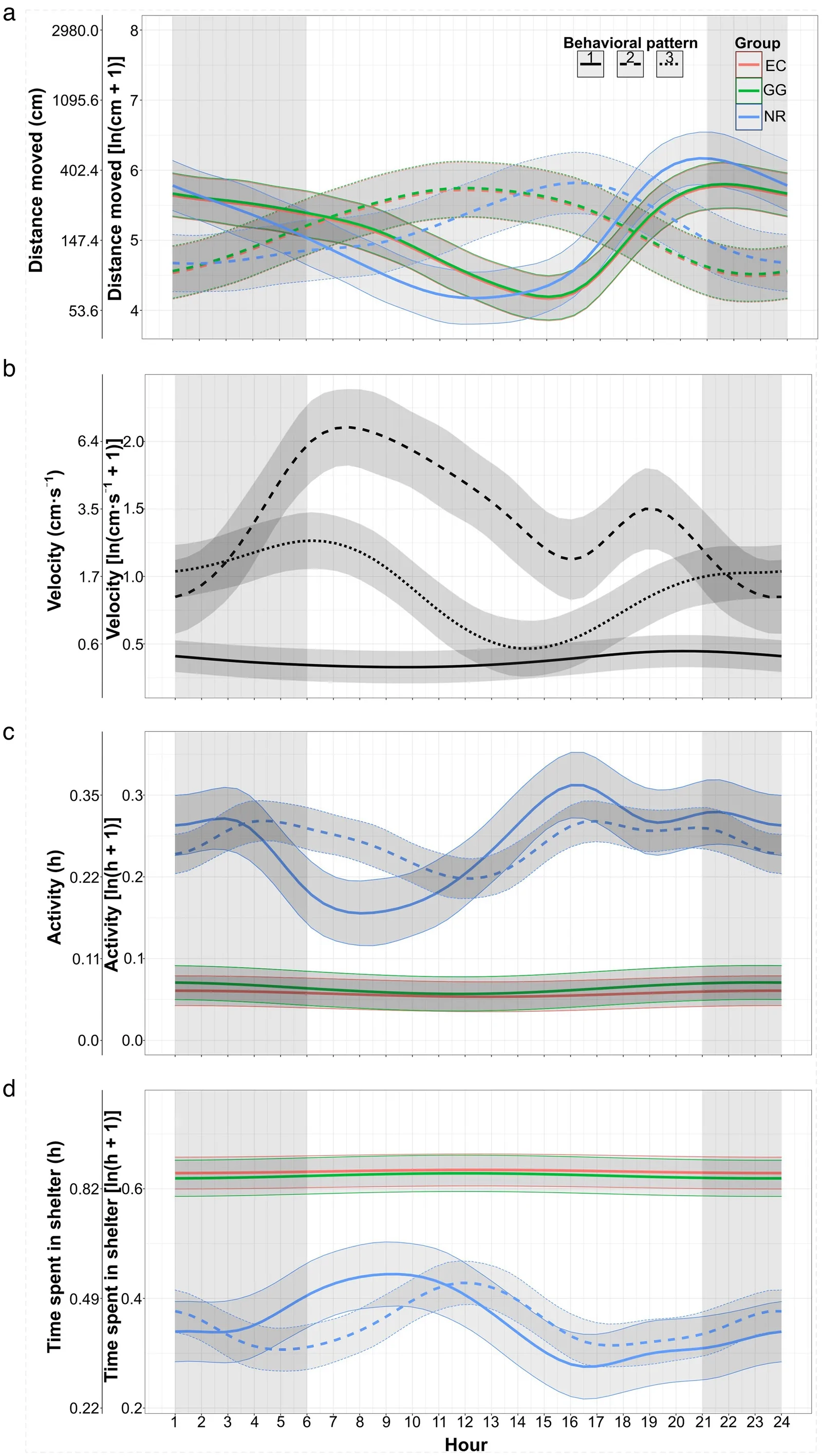
Experiment 2—shelter in arenas: fixed effects of the most parsimonious generalized additive mixed models (GAMMs) of marbled crayfish (*Procambarus virginalis*) behavioral responses. Distance moved, velocity, activity, and time spent in shelter were used as response variables in the GAMMs, diplayed in panels a, b, c, and d, respectively. Gray ribbons represent 95% confidence regions. Shaded areas represent dark period, normal area light period. The *y*-axes are shown as both raw values and log + 1-transformed. Abbreviations: EC, egg-carrying females; GG, glair gland females; and NR, non-reproductive females. For further details about behavioral patterns see Table 1.

In the presence of shelters, two main behavioral patterns emerged for the distance moved based on the most parsimonious LCLMMs (Supplementary Table S2 and Figure S3A): (1) “night-time locomotion” and (2) “day-time locomotion”. In EC, only 7% of individuals showed day-time locomotion, while in both GG and NR, 27% of individuals showed this day-time locomotion (Table 1 and Supplementary Figure S4A). Groups had significantly different temporal dynamics (*χ*^2^_1_ = 10.6, *P* = 0.001); however, the main effect of the group was not significant (*χ*^2^_2_ = 4.2, *P* = 0.12). NR showed different patterns compared with those of EC and GG which were identical (Figure 3A). For velocity, three main behavioral patterns were found (Supplementary Figure S3B): (1) “slow and constant velocity”, (2) “velocity with morning and evening peaks”, and (3) “velocity reduced day-time” (Table 1 and Figure 3B). There was a strong prevalence of slow velocity in the NR group (91% of individuals) compared with the GG and EC groups (with 27% of individuals and 50%, respectively). In addition, velocity-reduced day-time was not found in NR group, and there was no clear prevalence of this behavioral pattern in GG and EC (Table 1 and Supplementary Figure S4B). However, neither the main effect of the group (*χ*^2^_2_ = 5.2, *P* = 0.07), nor the differences in temporal dynamics of individual groups were significant (*χ*^2^_3_ = 0.98, *P* = 0.81). For activity, two distinct behavioral patterns were identified among marbled crayfish females (Supplementary Table S2 and Figure S3C): (1) “low activity” and (2) “activity reduced day-time” (Table 1 and Figure 3C). There was total prevalence of “low activity” in GG and EC (100% of females in both groups) compared with NR (only one female exhibited this trend; Table 1 and Supplementary Figure S3C). The main effect of group was highly significant (*χ*^2^_2_ = 70.8, *P* < 0.001) and the temporal dynamics between groups were also significantly different (*χ*^2^_3_ = 21.8, *P* < 0.001), indicating that the only NR females classified into “low activity” behavioral pattern by LCLMM showed rather more dynamic activity compared with other NR females (Figure 3C and Supplementary Figure S3C). Since the “time spent in shelter” was strongly negatively correlated with activity (*ρ* = −0.99), it reflected the same pattern found in activity across all crayfish individuals. Two behavioral patterns were identified (Supplementary Figure S3D): (1) “sheltering” (∼87% of time spent in shelter constantly during 24 h) and (2) “reduced sheltering” ∼36% of time was spent in shelter when the crayfish showed increased activity) (Table 1 and Figure 3D). Similarly as in activity, the temporal dynamics was significantly different between groups and the main effect of group was highly significant (*P* < 0.001).

### Experiment 3

**Figure 4:**
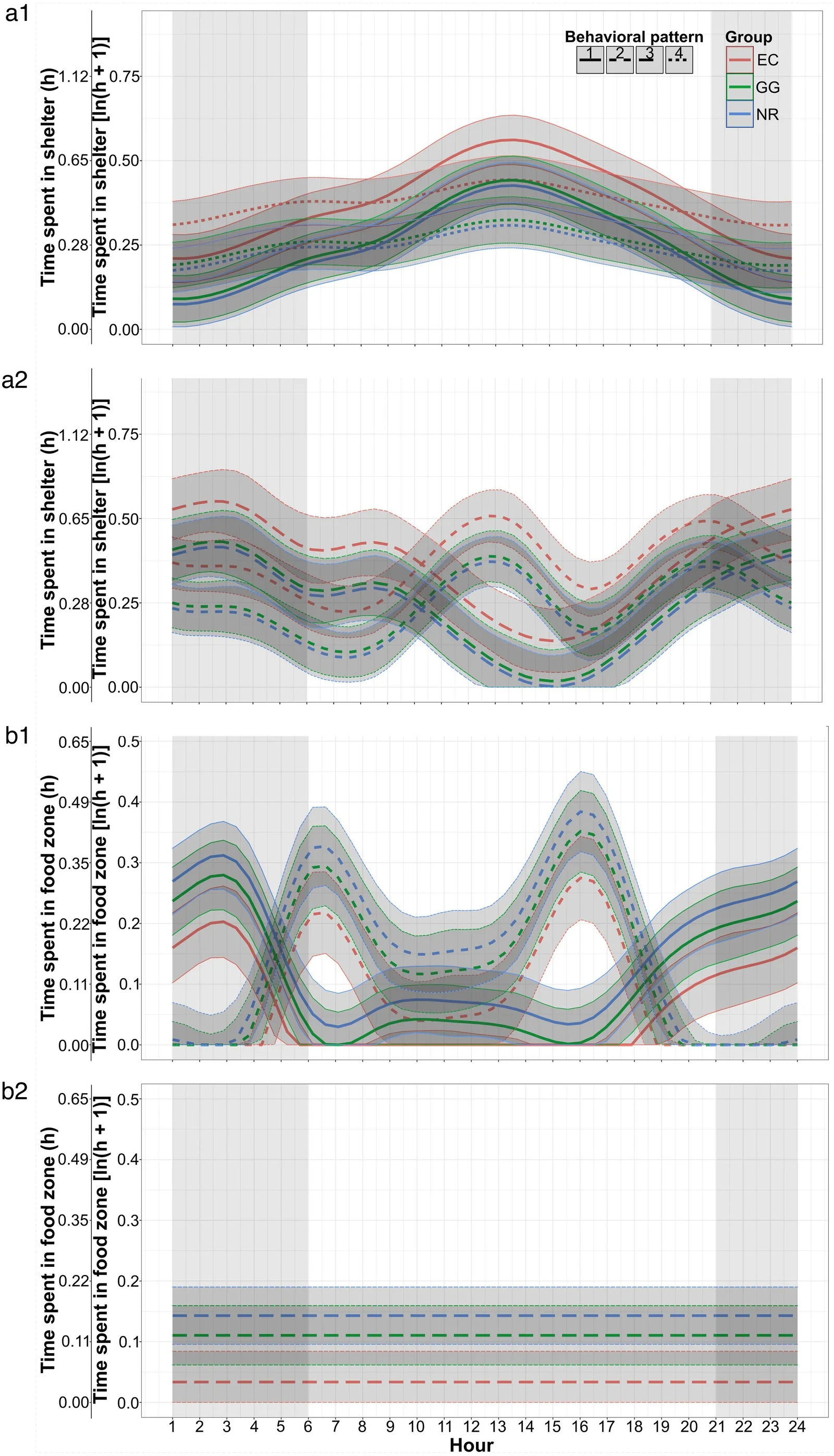
Experiment 3—shelter and food in arenas: fixed effects of the most parsimonious generalized additive mixed models (GAMMs) of marbled crayfish (*Procambarus virginalis*) behavioral responses. Both GAMMs, with time spent in shelter (A1 and A2) and with time spent in food zone (B1 and B2) as the responses, were divided into two subplots to provide better visualization. Gray ribbons represent 95% confidence regions. Shaded areas represent dark period, normal area light period. The *y*-axes are shown as both raw values and log + 1-transformed. Abbreviations: EC, egg-carrying females; GG, glair gland females; and NR, non-reproductive females. For further details about behavioral patterns see Table 1.

In the presence of shelter and food, four distinct behavioral patterns were identified among individual crayfish for time spent in the shelter across 24-h period (Supplementary Table S2 and Figure S5A): (1) “prominent day-time sheltering”, (2) “bimodal sheltering”, (3) “night and morning sheltering”, and (4) “less prominent day-time sheltering” (Table 1 and Figure 4A,B). Notably, “prominent day-time sheltering” was prevalent in the NR group, while the “less prominent day-time sheltering” pattern occurred more frequently in the EC group (Table 1 and Supplementary Figure S6A). A significant main effect of group was detected (*χ*^2^_2_ = 9.8, *P* = 0.007), although there were no significant differences between groups in temporal dynamics (*χ*^2^_3_ = 0.4, *P* = 0.97). Fitted values indicated higher shelter use in EC individuals compared with NR and GG (Figure 4A,B). Time spent in the food zone was not correlated with time spent in shelter (*ρ* = 0.23). Three distinct behavioral patterns emerged (Supplementary Figure S5B): (1) “night-time foraging”, (2) “bimodal day-time foraging activity”, and (3) “consistently low foraging activity” (Table 1 and Figure 4C,D). The latter behavioral pattern predominated in EC individuals and was more frequent in GG than NR, while “night-time foraging” was primarily seen in the NR group (Table 1 and Supplementary Figure S6B). The main effect of the group was again significant (*χ*^2^_2_ = 8.2, *P* = 0.01), though differences in temporal dynamics were not (*χ*^2^_3_ = 5.3, *P* = 0.15). Fitted values for time spent in the food zone were lowest in EC compared with NR and GG (Figure 4C,D).

## Discussion

Understanding the behavioral patterns of crayfish is important for studying their ecology. It is well known that when contrasted with the NR females, EC females largely reduce their overall activity, including foraging. Instead, they avoid interactions and preferably shelter. In that way, they reduce the energy expenditure and minimize the predation risk (Gutiérrez-Yurrita and Montes 1999; Linzmaier and Jeschke 2020), dedicating their focus to egg incubation, cleaning and fanning (Reynolds 2002; Vogt 2018). However, information on GG females is largely missing. In the present research, we studied how the reproductive stage of the marbled crayfish affects its behavioral patterns in general, as well as when provided with different critical resources. We found that GG females can show generally higher activity in arenas without shelter, when compared with the EC ones. However, GG females showed similar behavior as EC ones in the presence of shelter, in contrast to NR females which spent significantly less time in the shelter. On the other hand, in the presence of food, GG females again spent more time in the food zone than the EC group. In general, marbled crayfish show the highest activity in the morning and night (Farca Luna et al. 2009). That was also confirmed by our experiments; nevertheless, we found several other behavioral patterns among the females. Most crayfish have nocturnal habits (Davis and Huber 2007; Johnson et al. 2014; Hirsch et al. 2016); their locomotion increases at dusk, reaching a maximum at midnight, and continues until the early hours of the morning (Gherardi et al. 2002). This also applies to some congeneric species, such as the red swamp crayfish in nature (Gherardi et al. 2000). European native noble crayfish and narrow-clawed crayfish *Pontastacus leptodactylus* (Eschscholtz, 1823) were found to be more nocturnal than invasive spiny-cheek crayfish *Faxonius limosus* (Rafinesque, 1817) and signal crayfish *Pacifastacus leniusculus* (Dana, 1852) (Lozán 2000). However, when interacting with non-native crayfish, native crayfish can become more active during the day, making them more vulnerable to visual predators, as documented in noble crayfish and spiny-cheek crayfish (Musil et al. 2010). Thus, crayfish can show behavioral plasticity, altering their behavioral patterns under different circumstances (Reisinger et al. 2017). Indeed, some studies have discussed that a high degree of plasticity (e.g., switching of temporal niches) constitutes a strategy for maximizing survivability in response to environmental changes (Hut et al. 2012; van der Vinne et al. 2019). As demonstrated by our study, significant behavioral plasticity can appear among marbled crayfish individuals of a laboratory clonal stock origin, too.

The daily pattern is influenced by various factors, including refuge availability, predator avoidance, and orientation mechanisms; additionally, physiological and biological traits like starvation, molting, and reproduction play significant roles. A similar pattern of activity has been observed in invasive crayfish (Robinson et al. 2000; Barbaresi and Gherardi 2001). For example, in spring, red swamp crayfish were more active during the day-time (Gherardi et al. 2002). During the breeding period, migrations have been recorded in female virile crayfish *Faxonius virilis* (Hagen, 1870) (Hazlett et al. 1979) and both sexes of spiny-cheek crayfish (Buřič et al. 2009a; Buřič et al. 2009b). Nonetheless, it should be noted that laboratory setups, which are inevitably simplified compared to natural conditions, may also affect behavioral patterns. This was found, for example, in red swamp crayfish, which showed diurnal activity in laboratory conditions (Gherardi et al. 2000). In marbled crayfish, information about behavioral patterns comes mostly from laboratory conditions. For example, Farca Luna et al. (2009, 2010) studied its behavioral patterns in the laboratory and reported shifts in their activity according to the dominance hierarchy.

Our study suggests that in the absence of resources, there was no difference in the temporal dynamics of activity and velocity among tested groups; however, GG females spent more time moving, when compared with both NR and EC ones. This prominent activity of GG females seems surprising, as one could expect they will gain intermediate values between NR and EC ones, considering that crayfish reproduction is a cyclic process. The explanation of elevated activity in GG females can be seen from several perspectives. In crayfish species, both sexes are known to elevate their activities during the mating period when searching for partners (Hazlett et al. 1979; Buřič et al. 2009a, b). Even though marbled crayfish is a parthenogenetic species in which males are absent, this does not mean its females are not influenced by identical triggers, such as hormonal changes during final egg maturation, altering their behavior (Vogt 2015, 2018; Harlıoğlu et al. 2017), eventually searching for males. Final egg maturation is an energetically and nutritionally demanding process (Harlıoğlu and Farhadi 2017; He et al. 2021; Das et al. 2024a, 2024b). Therefore, one might assume that despite the absence of food in Experiment 1, GG females tend to forage more than other groups. Another contributing factor could be that EC females largely reduce their food intake during an extended time of incubation and early juvenile development, forcing them to maximize foraging activity just before oviposition, coinciding with their GG stage. Similarly, the search for a suitable shelter is another important motivation of females just before spawning (Buřič et al. 2009a; Hirsch et al. 2016), as it significantly increases their survival (e.g., predator avoidance), and hence reproduction success.

When the shelter was available in the arena, the three reproductive stages differed more, with EC and GG females primarily hiding there, unlike NR ones. However, this pattern was changed when food was also present. Despite foraging activity being the most pronounced in NR females, both EC and GG ones also showed foraging, and ultimately spent less time in the shelter. In nature, shelters play an important role in hiding from predators and other, both intra- and inter-specific competitors (Gherardi and Cioni 2004; Matsuzaki et al. 2012), and provide refuge from extreme fluctuations in abiotic conditions, significantly affecting the fitness of individuals (Holdich 2002; Gherardi and Daniels 2004; Kouba et al. 2016). As such, crayfish seek shelters when they are less active (Bubb et al. 2009) and reduce their activity in the presence of a shelter (Aréchiga et al. 1993; Farca Luna et al. 2009). We found a different temporal pattern in the use of the shelter by the different reproductive stages when only the shelter was provided. Nonetheless, we found more variable sheltering time amongst groups when food was also provided, pointing to differences in energy needs between the three reproductive stages. Indeed, energy and nutrients must be allocated between physiological functions such as body growth, reproduction, and maintenance (Helm et al. 2017). While EC crayfish females have already invested their energy resources and reduced their feeding (Sheppard et al. 2024), NR females primarily need to sustain themselves and establish territories (Linzmaier and Jeschke 2020), and GG females require a significant amount of energy for final eggs maturation as well as reserves for upcoming incubation. Therefore, GG females become more active to maximize their energy intake, and also explore new areas (Gutiérrez-Yurrita and Montes 1999; Das et al. 2024a), a general pattern seen even in empty arenas without critical resources provided.

Overall, our results provide the first comprehensive behavioral evidence that marbled GG females show a peculiar behavior distinct from that of EC females. Therefore, future research should consider these differences in their designs. Our results also highlighted the importance of considering the time in experimental setups involving marbled crayfish due to daily behavioral variations. It appears that the timing of experiments should be considered when comparing the observed findings such as behavioral performances between different studies or experiments on this species. We believe our findings can be largely extrapolated to the other crayfish species.

## Supplementary Material

zoaf062_Supplementary_Data

## Data Availability

The data and R script used for the data analysis are available at: https://github.com/ParideBalzani/das.
