# Supplementary material for: Reproductive stage affects the daily behavioral patterns of parthenogenetic marbled crayfish
Source: Curr Zool. 2025 Sep 17;72(4):523–35. doi: 10.1093/cz/zoaf062 (PMC13500160; doi:10.1093/cz/zoaf062)
Supplement: Supplementary file 1 [file zoaf062_supplementary_data.docx]

**Reproductive status affects the daily activity patterns of parthenogenetic marbled crayfish**

**Supplementary Materials**

Koushik Das (ORCID: 0000-0002-9128-3952), Paride Balzani (ORICD: 0000-0003-1549-7139), Davinder Kaur (ORCID: 0000-0001-5604-6516), Jan Kubec (ORCID: 0000-0001-8212-1962), Miloš Buřič (ORCID: 0000-0003-2220-5579), Antonín Kouba (ORCID: 0000-0001-8118-8612), Marek Let (ORCID: 0000-0002-5494-8222)*

Faculty of Fisheries and Protection of Waters, South Bohemian Research Centre for Aquaculture and Biodiversity of Hydrocenoses, University of South Bohemia in České Budějovice, Zátiší 728/II, 389 25 Vodňany, Czech Republic

*Corresponding author: [mlet@frov.jcu.cz](mailto:akouba@frov.jcu.cz) (Marek Let)

**Table S1.** Cephalothorax length (CL, mm) and body weight (W, g) of marbled crayfish (*Procambarus virginalis*) individuals used in the three experiments. Values are presented as mean ± standard deviation. One-way ANOVA following log-transformation of the response variables was used to compare differences among reproductive groups within each experiment. No significant differences among groups were found in CL and W (*p* > 0.05). NR—non-reproductive females; GG—females with developed glair glands; EC—egg-carrying females.

| Experiment | Groups (n) | CL (mm) | *p*-value | W (g) | *p*-value |
| --- | --- | --- | --- | --- | --- |
| 1 Empty arena | NR (22) | 19.59 ± 1.03 | 0.85 | 1.94 ± 0.21 | 0.90 |
|  | GG (22) | 19.73 ± 1.16 |  | 1.97 ± 0.30 |  |
|  | EC (22) | 19.78 ± 1.03 |  | 1.99 ± 0.37 |  |
| 2 Shelter | NR (16) | 19.90 ± 1.58 | 0.35 | 1.95 ± 0.66 | 0.44 |
|  | GG (16) | 19.17 ± 1.28 |  | 1.70 ± 0.32 |  |
|  | EC (16) | 19.28 ± 1.52 |  | 1.84 ± 0.55 |  |
| 3 Shelter + food | NR (16) | 19.14 ± 1.91 | 0.64 | 1.82 ± 0.51 | 0.49 |
|  | GG (16) | 18.69 ± 1.85 |  | 1.70 ± 0.62 |  |
|  | EC (16) | 19.29 ± 1.61 |  | 1.97 ± 0.69 |  |

**Table S2.** Values of Baesian information criterion (BIC) for 1-class to 5-class linear latent class linear mixed models (LCLMMs) used for each log+1-transformed response variable and each experiment. For each response variable and experiment, the lowest BIC value is bolded.

| Experiment | Response variable | 1-class LCLMM | 2-class LCLMM | 3-class LCLMM | 4-class LCLMM | 5-class LCLMM |
| --- | --- | --- | --- | --- | --- | --- |
|  |  | BIC | | | | |
| 1 Empty arena | Distance moved | 3970.8 | **3916.0** | 3917.2 | 3938.7 | 3957.2 |
|  | Velocity | –100.1 | **–109.3** | –94.5 | –82.3 | –79.7 |
|  | Activity | –3426.6 | –3499.9 | **–3508.7** | –3501.5 | –3500.3 |
| 2 Shelter | Distance moved | 2910.6 | **2878.9** | 2889.4 | 2901.8 | 2908.6 |
|  | Velocity | 1790.3 | 1759.5 | **1757.9** | 1758.0 | 1771.5 |
|  | Activity | –2712.8 | **–2749.1** | –2732.5 | –2722.2 | –2704.3 |
|  | Time spent in shelter | –1838.3 | **–1882.9** | –1868.5 | –1853.1 | –1835.6 |
| 3 Shelter + food | Time spent in shelter | –489.3 | –546.7 | –548.6 | **–565.2** | –549.8 |
|  | Time spent in food zone | –1014.7 | –1134.3 | –1253.1 | **–1234.4** | –1232.3 |

**Fig. S1.** The most parsimonious latent class linear mixed models (LCLMMs). A—log+1 distance moved, B—log+1 velocity, and C—log+1 activity (time spent in locomotion) of marbled crayfish (*Procambarus virginalis*).

**Fig. S2.** The most parsimonious generalized additive mixed models (GAMMs) displaying random effects of each individual marbled crayfish (*Procambarus virginalis*). A—log+1 distance moved, B—log+1 velocity, and C—log+1 activity (time spent in locomotion). The black lines represent additional locally estimated scatterplot smoothing (LOESS) lines. Abbreviations: EC—egg-carrying females, GG—glair gland females, and NR—non-reproductive stage females.

**Fig. S3.** The most parsimonious latent class linear mixed models (LCLMMs). A—log+1 distance moved, B—log+1 velocity, C—log+1 activity (time spent in locomotion), and D—log+1 time spent in shelter of marbled crayfish (*Procambarus virginalis*).

**Fig. S4.** The most parsimonious generalized additive mixed models (GAMMs) displaying random effects of each individual marbled crayfish (*Procambarus virginalis*). A—log+1 distance moved, B—log+1 velocity, and C—log+1 activity (time spent in locomotion), and D—log+1 time spent in shelter. The black lines represent additional locally estimated scatterplot smoothing (LOESS) lines. Abbreviations: EC—egg-carrying females, GG—glair gland females, and NR—non-reproductive stage females.

**Fig. S5.** The most parsimonious latent class linear mixed models (LCLMMs). A—log+1 time spent in shelter and B—log+1 time spent in food zone in marbled crayfish (*Procambarus virginalis*).

**Fig. S6.** The most parsimonious generalized additive mixed models (GAMMs) displaying random effects of each individual marbled crayfish (*Procambarus virginalis*). A—log+1 time spent in shelter and B—log+1 time spent in food zone. The black lines represent additional locally estimated scatterplot smoothing (LOESS) lines. Abbreviations: EC—egg-carrying females, GG—glair gland females, and NR—non-reproductive stage females.
